# Comparative analysis of cardio-cerebrovascular complications in immigrants and native-born Koreans with diabetes: Risk factors and perspectives

**DOI:** 10.1371/journal.pone.0263046

**Published:** 2022-04-29

**Authors:** Hyemin Cho, Sohyun Jeoung, Cinoo Kang, Sunmee Jang

**Affiliations:** 1 College of Pharmacy and Gachon Institute of Pharmaceutical Sciences, Gachon University, Incheon, Republic of Korea; 2 Health Insurance Research Institute, National Health Insurance Service, Wonju, Republic of Korea; 3 Marcus Institute for Aging Research, Hebrew SeniorLife, Boston, MA, United States of America; 4 Department of Medicine Beth Israel Deaconess Medical Center and Harvard Medical School, Boston, MA, United States of America; 5 Department of Biostatics and Epidemiology Graduate School of Public Health, Seoul National University, Seoul, Republic of Korea; Chiang Mai University, THAILAND

## Abstract

**Background:**

Given the rapidly increasing number of immigrants, it is crucial to address health care issues involving immigrants to facilitate their safe and secure settlement. Especially for common chronic diseases, such as diabetes, immigrants face more complex obstacles to manage their chronic conditions than do native-born residents. Therefore, we aimed to assess differences in the incidence and associated risk factors of cardio-cerebrovascular (CCV) complications of immigrants compared with native-born Koreans with diabetes.

**Methods:**

Immigrants and native-born Koreans who had new diagnosis of diabetes and simultaneously received anti-diabetic prescriptions in 2012 were defined by using Korean National Health Insurance Claim Database(KNHICD). CCV complications were assessed at a 3-year follow-up from the index date. We assessed differences in the CCV complications and risk factors using multiple cox regression models.

**Results:**

In total, 4,008 patients (668 of immigrants and 3,340 of native-born Koreans) who had newly diagnosed diabetes and simultaneously received anti-diabetic prescriptions in 2012 were selected. Immigrants with diabetes were at a 1.39 times higher risk of having CCV complications than native-born Koreans with diabetes (95% CI: 1.021–1.881). Patients who had a usual sources of care (USC) presented a significantly reduced risk of cardio-cerebrovascular complication (HR: 0.452; 95% CI: 0.342–0.598) in both immigrants and native Koreans. In subgroup analysis in immigrants, patients having USC showed decreased risk of CCV incidence (HR: 0.35, 95% CI: 0.175–0.703), whereas >60 years old and Charlson comorbidity index (CCI) score >1 presented increased risk of CCV complications.

**Conclusion:**

Immigrants with diabetes have a higher risk of CCV complications than native-born Koreans with diabetes. However, having a USC significantly decreased the risk of CCV complications. Therefore, the utilization of USC will benefit to reduce diabetic complications in immigrants as well as reduction of overall health care cost burden, it would be necessary to implement USC in diabetes care at the initial disease stage.

## Introduction

Globally, the rate of immigration has been increasing consistently. Korea is a latecomer in the history of immigration, but the number of immigrants in Korea has dramatically increased in recent years [1]. Based on the report by UN DESA, the number of immigrants reached 270 million in 2019, an increase of 50 million since 2010 [2]. For the immigrants in Korea, the number has been doubled to 2.1 million in 2017 from 1 million in 2007 [3]. Given the rapidly increasing number of immigrants, it is crucial to address health care issues involving immigrants to facilitate their safe and secure settlement. The health status of immigrants in the early period of immigration is generally assumed to be fairly high, as reflected by the well-known principle of the ‘healthy immigrant effect’ [4,5]. However, when health problems occur during acculturation, several obstacles driven by language barrier, financial problems, cultural and social differences, and differences in health care systems hinder immigrants from timely utilization of relevant health care services, posing an additional health care burden both to immigrants themselves and to society as a whole [6–11].

Especially for common chronic diseases, such as diabetes, which require life-long utilization of pharmaceutical and medical care, immigrants face more complex obstacles to manage their chronic conditions than do native-born residents [11–13]. According to previous studies, higher rates of diabetes incidence, accompanying complications and mortality were observed in immigrants than in native-born residents. [12,14–17]. In a study in the Netherlands, mortality attributable to diabetes was 3–4 times higher among immigrants than among native-born Dutch [18]. In the UK, complications of diabetes occurred more frequently and deaths from coronary heart disease were twice higher in 30- to 64-year-old immigrants than in native-born residents of the same age [19]. However, it has been well documented that the complications of diabetes can be reduced by regularly taking anti-diabetic medications, which contributes to an overall reduction of medical costs [20–23]. When patients make regular clinical visits as appropriate, physicians become well aware of their disease, treatment, and compliance history; this enables physicians to help patients prevent the aggravation of disease in advance, resulting in reduced medical costs [24,25]. Therefore, it is important to provide immigrants with a health care environment in which they can receive regular and consistent treatment.

In this context, we aimed to assess differences in the incidence and associated risk factors of cardio-cerebrovascular (CCV) complications between immigrants and native-born Koreans with diabetes. Furthermore, we aimed to evaluate the impact of having a usual source of care (USC) for diabetes management in immigrants and thereby to provide fundamental knowledge to support health care policy decision-making.

## Methods

### Design and study population

We utilized the Korean National Health Insurance Claim Database (KNHICD) as a source to establish a cohort comprising immigrants and Korean controls. The KNHICD, which is a mandatory national health insurance system, is composed of claims data for recipients of National Health Insurance coverage of 97% of the population and for recipients of Medical Aid coverage of the remaining 3% of the population. Except for uninsured medical care, such as plastic surgery or off-label use, most treatment-related data (International Classification of Diseases, 10th Revision [ICD-10] diagnosis codes, medical costs, characteristics of clinics, and prescription information) can be retrieved from the KNHICD. Additionally, an item on eligibility for insurance coverage provides the immigration status of foreigners residing in Korea, enabling us to retrieve information for various categories of immigrants. We selected foreigners who had diabetes ICD-10 codes (E11, E12, E13, E14) as primary to the fifth level diagnosis code and had prescriptions for anti-diabetic medications in 2012 [26]. To ascertain true diabetes patients, patients who had two and more outpatient clinic visits, or one and more inpatient department visits were included. We excluded patients having ICD-10 codes for diabetes in previous years to include only new-onset patients with antidiabetics in 2012 since diabetes complications are related to the period of diabetes [27,28]. Native-born Korean patients with diabetes were matched as controls at a 1:5ratio (1: immigrants, 5: native-born Koreans) to overcome power issue [29]. The matching was performed via propensity matching score calculated using sex, age (allowing a 1-year range), and the Charlson Comorbidity Index (CCI; 0, 1, 2, 3 and over). CCI was developed as a weighted index to predict risk of death within 1 year of hospitalization for patients with specific comorbid conditions [30,31]. If patients have comorbid condition with high mortality risk, the condition gets higher weights from 1-year mortality hazard ratio from a Cox proportional hazards model. These weights were summed to produce the Charlson comorbidity score. CCI was basically developed using hospital medical records. Since CCI calculation algorithm using ICD-10 by Quan et al [32] showed the highest predictability, Korean researchers use Quan et al’s algorithm and calculate CCI score using KCD5 (ICD-10 in Korea) in administrative data (health care claim data) [33,34]. CCI score is usually classified into 4 categories (0, 1, 2, and 3+) but it can be reclassified by the researchers based on the overall distribution. [33]. In this study, we also calculated the CCI based on the ICD-10 codes and weights produced by the previous study [31] and classified the scores into 0,1,2, and 3+.

### Outcome variables

CCV complications were assessed at a 3-year follow-up from the index date (the first diagnosis date in both immigrants and Koreans with diabetes). CCV complications were defined by ICD-10 codes as follows: 1) cardiovascular complications: angina pectoris (I20.x), myocardial infarction (MI; I21.x, I22.x, I23.x I25.2), other ischemic heart disease (I24.x), other chronic ischemic heart disease (I25.x [except I25.2]), heart failure (I50.x), atherosclerosis (I70.x), and aortic aneurysm or dissection (I71.x); and 2) cerebrovascular complications: transient ischemic attack (TIA; G45.x), stroke (I63.x, I65.x, I66.x). To select patients with true complications, we had stringent criteria as such; patients who had the above mentioned codes as primary diagnosis codes and had records of 2 or more days of inpatient department admission, or 2 outpatient department visits within 3 months, or one or more procedure code for percutaneous coronary intervention (PCI; M6551, M6552, M6561, M6562, M6563, M6564, M6572) or coronary artery bypass graft (CABG; O1641, O1642, O1647, OA641, OS647).

### Independent variables

Risk factors potentially affecting the incidence of CCV complications in patients with diabetes were analyzed as covariates. Age (<60 years vs. ≥60 years), sex, insurance type (National Health Insurance vs. Medical Aid), residential area (urban vs. rural area), number of household members (single vs. non-single) and the presence of a physical disability in 2012 were assessed. For residential area, they were defined based on Korean regional district classification such as Si (medium size city), Gu (large city), and Gun (rural area). Insurance status was categorized as National Health Insurance (NHI) and Medical Aid. Medical Aid is a service received by low-income individuals (30% of the median income or below), and qualified Medical Aid beneficiaries make no or little copayments for medical treatment. Insurance premium tier was also included to assess the economic factor given insurance premium tier is classified to 20 strata. 0 represents the lowest and 20 represents the highest income levels. We classified the tier to 0–4 and 5–20 as comparators.

Based on the previous study [35], the number of antidiabetic agents is associated with the diabetic severity, the number of anti-diabetic medications (1 vs. 2 or more) was included in covariate analysis. The insulin prescription at the first diabetes diagnosis was included as well to assess the diabetes severity.

The type of medical institution that issued each prescription for anti-diabetic medication (tertiary hospital, secondary hospital, primary hospital [clinic], or public health center) were assessed as factors reflecting the severity of diabetes. Tertiary hospitals were defined as having more than 20 medical departments (including 9 essential medical departments) and being qualified to provide educational training to medical residents. Secondary hospitals were defined as having 200–500 beds with more than 4 medical departments and performing more complicated surgery than primary hospitals. Clinics are usually run by a single physician, with a specific medical focus, and public health centers are government-supported health care centers that provide basic health care support (Medical service Act No.16555 (2019)) [36].

Comorbidities were defined as including other diseases associated with diabetes and CCV complications, including hypertension (ICD-10 I10, I11, I12, I13, and I15) and hyperlipidemia (ICD-10 E78). Their ICD-10 codes were recorded within 1 year of the index date as primary diagnosis codes. Patients were matched by their CCI score, which was categorized as 0, 1, 2, or 3+, with a higher number indicating severe disease status. The medication possession ratio (MPR) was used as a compliance index for anti-diabetic medications. The MPR is calculated as the ratio of days that a medication is prescribed divided by the number of follow-up days (study period) [37]. We measured 1-year MPR. The MPR threshold for high drug compliance was set at 80%, with ≥80% meaning high compliance and <80% meaning low compliance [38,39]. For patients in whom complications occurred, the MPR was calculated until the date of the complication event. Since Korea doesn’t have primary care physician (PCP) in health care system, we utilized the term Usual Source of Care (USC) to evaluate the continuity of care USC contains primary care institutions as well as secondary and tertiary hospitals, which is distinctive from PCP. Based on the previous study examining USC has high possibility to represent the indices of preventative and chronic care in general population [40]. Therefore, we hypothesized that USC variable might be associated with CCV incidences. For operational definition in this study, if patients used only 1 medical institution for diabetes care within 1 year of the index date, then they were categorized as having a USC.

### Statistical analysis

The characteristics of immigrants and native-born Koreans were compared using the chi-square test for categorical variables and the t-test for continuous variables. The risk of diabetic CCV complications was assessed using a Cox proportional hazards model adjusting for multiple covariates (age, sex, MPR, USC, number of anti-diabetic medications in the first prescription, insurance type, residential area, number of household members, disability status, hypertension, hyperlipidemia, and type of medical institution). The results are presented as hazards ratio (HRs) with 95% confidence intervals (CIs). All statistical analyses were performed using SAS version 9.3 (SAS Institute Inc., Cary, NC) and the two-tailed level of statistical significance was defined as p<0.05.

### Ethics statement

This study was reviewed and approved by the Institutional Review Board (IRB) of Gachon University, according to IRB regulations (IRB No:1044396-201710-HR-169-01).

## Results

**Fig 1:**
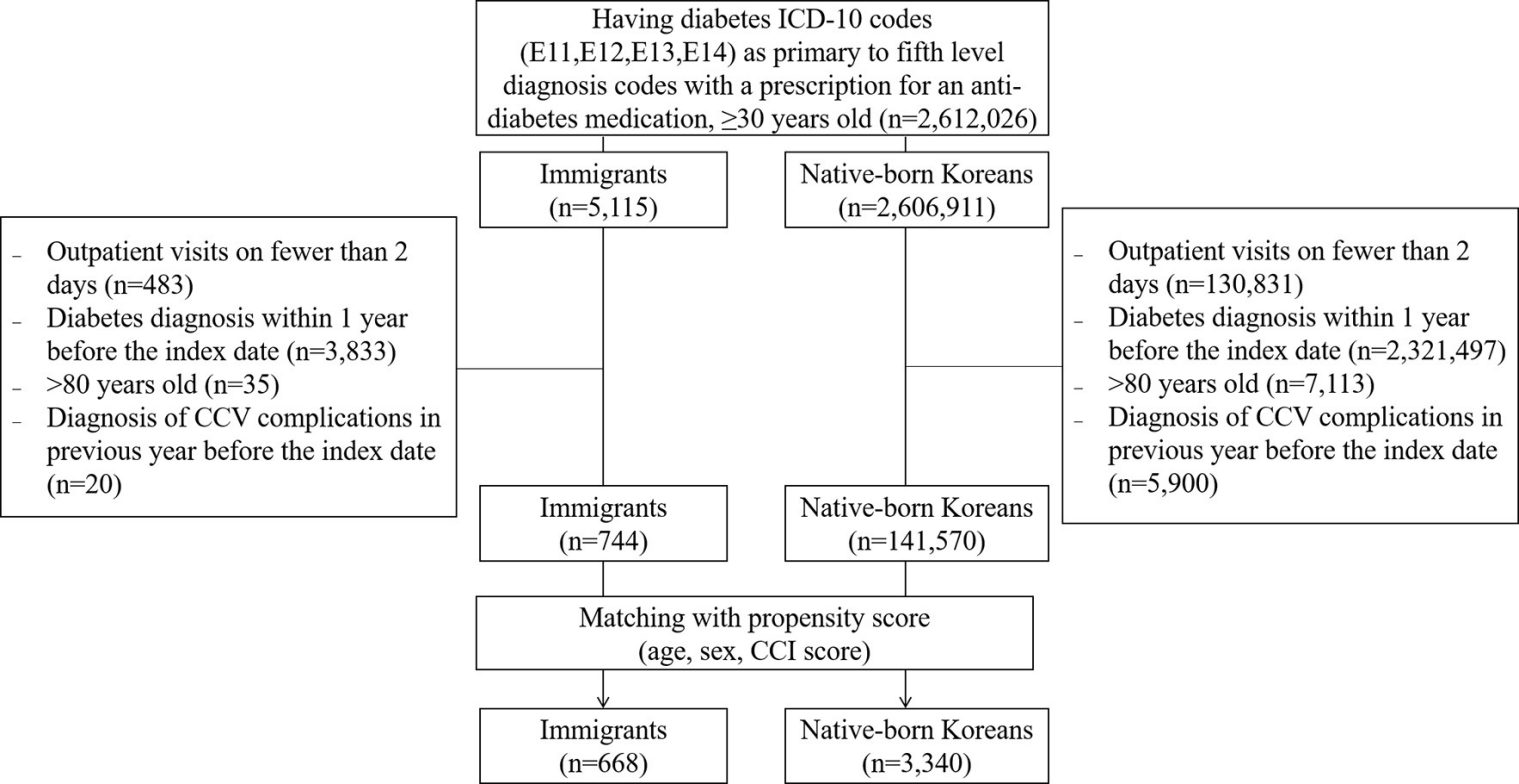
Flow diagram of selection of study population.

In total, 668 immigrants and 3,440 native-born Koreans who had newly diagnosed diabetes and simultaneously received anti-diabetic prescriptions in 2012 were selected. Since the history of immigration in Korea is rather short and out inclusion criteria was stringent to only include pure new-onset diabetes, only 30- to 79-year-old immigrants were selected in the cohort selection process. To overcome the power issue, we applied 1.5 matching ratio (1: immigrants, 5: native-born Koreans) resulting in 668 immigrants and 3,340 native-born Koreans (Fig 1).

There were slightly more men (51.7%) than women, and their mean age was 55 years (standard deviation [SD] 11.2 years). The overwhelming majority (81.8%) of patients had the lowest CCI level (0).

The vast majority of both immigrants (95.2%) and Koreans (88.4%) resided in urban areas, but urban residence was significantly more common among immigrants (p<0.0001). Insurance type was not significantly different between the two groups (p = 0.4669). Immigrants (38.8%) more frequently resided alone than Koreans (22.3%) (p<0.0001). The drug compliance index (MPR) was significantly lower in immigrants (69% [SD 29.2%] vs. 72% [SD 30.2%]; p = 0.0197). The comorbid conditions of hypertension and hyperlipidemia were less common in immigrants than in Koreans (hypertension: 18.1% vs. 31.6%; hyperlipidemia: 2.8% vs. 5.2%; p<0.0001).

The medical institutions that issued the first prescription for an anti-diabetic medication had broadly similar trends in both immigrants and Koreans, with most prescriptions issued at clinics and public health centers (immigrants: 66.0% vs. Koreans: 63.8%), followed by secondary hospitals, primary hospitals, and tertiary hospitals; the difference between the two groups for this parameter was not significant (p = 0.141). A higher proportion of Koreans had a USC (41.7%) than immigrants (36.1%) (p = 0.0075). The number of anti-diabetic medications at the first prescription was not significantly different between the groups, but a higher proportion of Koreans had a disability (p < .0001). Immigrants who had insulin prescription at the first diagnosis were 8.9%, but it was 9.9% for Koreans, which were not significantly different. Naturalized immigrants comprised the majority of immigrants (56.1%), followed by permanent residents (26.7%) and marriage immigrants (17.2%) (Table 1).

**Table 1:** Characteristics of immigrants and native-born Koreans with diabetes.

Category		Total (n = 4,008)		Immigrants (n = 668)		Native-born Koreans (n = 3,340)		p-value
		N	%	N	%	N	%	
Sex	Male	2,072	51.70	346	51.80	1,726	51.68	0.9549
	Female	1,936	48.30	322	48.20	1,614	48.32	
Age	<60 years	2,728	68.06	454	67.96	2,274	68.08	0.9997
	≥60 years	1,280	31.94	214	32.04	1,068	31.92	
	Mean ±SD	55 ± 11.19		55 ± 11.2		55 ± 11.19		0.9055
CCI	0	3,280	81.84	545	81.59	2,735	81.89	0.9687
	1	585	14.60	97	14.52	488	14.61	
	2	87	2.17	16	2.40	71	2.13	
	3+	56	1.40	10	1.50	46	1.38	
Residential area	Urban area	3,590	89.57	636	95.21	2,954	88.44	< .0000
	Rural area	418	10.43	32	4.79	386	11.56	
Insurance type	NHI	3,797	94.74	629	94.16	3,168	94.85	0.4669
	Medical Aid	211	5.26	39	5.84	172	5.15	
Insurance premium tier	0–4	946	23.6	200	29.94	746	22.34	< .0001
	5–20	3,062	76.4	468	70.06	2,594	77.66	
Number of household members	Self (1)	1,005	25.07	259	38.77	746	22.34	< .0001
	Non-self (>1)	3,003	74.93	409	61.23	2,594	77.66	
MPR	<80%	1,735	43.29	330	49.40	1,405	42.07	0.0005
	≥80%	2,273	56.71	338	50.60	1,935	57.93	
	Mean ±SD	72±30.09		69±29.24		72±30.23		0.0197
Comorbidities	Hypertension	1175	29.32	121	18.11	1054	31.56	< .0001
	Hyperlipidemia	192	4.79	19	2.84	173	5.18	0.0099
	Total	1332	33.23	137	20.51	1195	35.78	< .0001
Medical institution that first issued anti-diabetic prescription	Tertiary hospital	234	5.84	29	4.34	205	6.14	0.1406
	Secondary hospital	669	16.69	119	17.81	550	16.47	
	Primary hospital	534	13.32	79	11.83	455	13.62	
	Clinic/ Public health center	2571	64.15	441	66.02	2130	63.77	
USC	No	2376	59.28	427	63.92	1949	58.35	0.0075
	Yes	1632	40.72	241	36.08	1391	41.65	
Number of anti-diabetic medications	1	2879	71.83	462	69.16	2417	72.37	0.0929
	≥2	1129	28.17	206	30.84	923	27.63	
Insulin prescription at the first diagnosis	No	3617	90.24	608	91.02	3009	90.09	0.4605
	Yes	391	9.76	60	8.98	331	9.91	
Disability	No	3710	92.56	652	97.60	3058	91.56	< .0001
	Yes	298	7.44	16	2.40	282	8.44	
Immigration status	Permanent resident	-	-	178	26.65	-	-	-
	Marriage immigrant	-	-	115	17.22	-	-	
	Naturalized	-	-	375	56.14	-	-	

*CCI; Charlson Comorbidities Index, NHI; National Health Insurance, MPR; Medication possession rate, USC; Usual source of care.

Table 2 shows that immigrants with diabetes were at a 1.39 times higher risk of having CCV complications than native-born Koreans with diabetes (HR: 1.386, 95% CI: 1.021–1.881). About two times higher risk of CCV complications were presented in ≥60 year old group compared to < 60 year old group (HR: 2.324; 95% CI: 1.812–2.98). Subjects who had a USC showed considerably decreased risk of CCV by 54% compared with the subjects not having a USC (HR: 0.452; 95% CI: 0.342–0.598). Subjects having hypertension and hyperlipidemia presented increased risk by1.5 times and 1.7 times, respectively (Hypertension HR: 1.546, 95% CI: 1.196–1.999, hyperlipidemia HR: 1.749. 95% CI: 1.115–2.743). Tertiary hospital was associated with an increased risk of CCV complications compared to Clinic/public health center (HR: 2.029; 95% CI: 1.311–3.141).

**Table 2:** Contributing factors for cardio-cerebrovascular complications in all subjects including immigrants and native-born Korean with diabetes.

Parameter		Reference	CCV complications	
			HR	95% CI
**Immigrants**		**Native-born Koreans**	**1.386**	**1.021–1.881**
Male		Female	1.114	0.877–1.415
**≥ 60 years**		**< 60 years**	**2.324**	**1.812–2.98**
CCI score: 1		CCI score: 0	1.201	0.879–1.64
CCI score: 2		CCI score: 0	1.585	0.872–2.88
CCI score: 3+		CCI score: 0	1.468	0.676–3.188
MPR (continuous)			1.001	0.997–1.005
**USC (yes)**		**No**	**0.452**	**0.342–0.598**
Number of anti-diabetic medication at the first diagnosis Anti-diabetic medications: ≥2		Anti-diabetic medications: < 2	1.079	0.814–1.429
Insulin prescription at the first diagnosis		non-insulin prescription	0.836	0.539–1.298
Urban area		Rural area	0.973	0.676–1.402
Number of household members: self		Number of household members: not self	0.952	0.72–1.258
NHI		Medical aid	0.847	0.51–1.406
Insurance premium tier 5–20		Insurance tier 0–4	1.095	0.794–1.512
Disability		Non-Disability	1.314	0.903–1.913
**Hypertension (yes)**		**No**	**1.546**	**1.196–1.999**
**Hyperlipidemia (yes)**		**No**	**1.749**	**1.115–2.743**
**Medical institution visited at the first diagnosis**	**Tertiary hospital**	**Clinic/public health center**	**2.029**	**1.311–3.141**
	Secondary hospital	Clinic/public health center	1.31	0.947–1.812
	Primary hospital	Clinic/public health center	1.035	0.72–1.489

*CCV; Cardio-cerebrovascular, CCI; Charlson Comorbidities Index, MPR; Medication possession rate, USC; Usual source of care.

**Fig 2:**
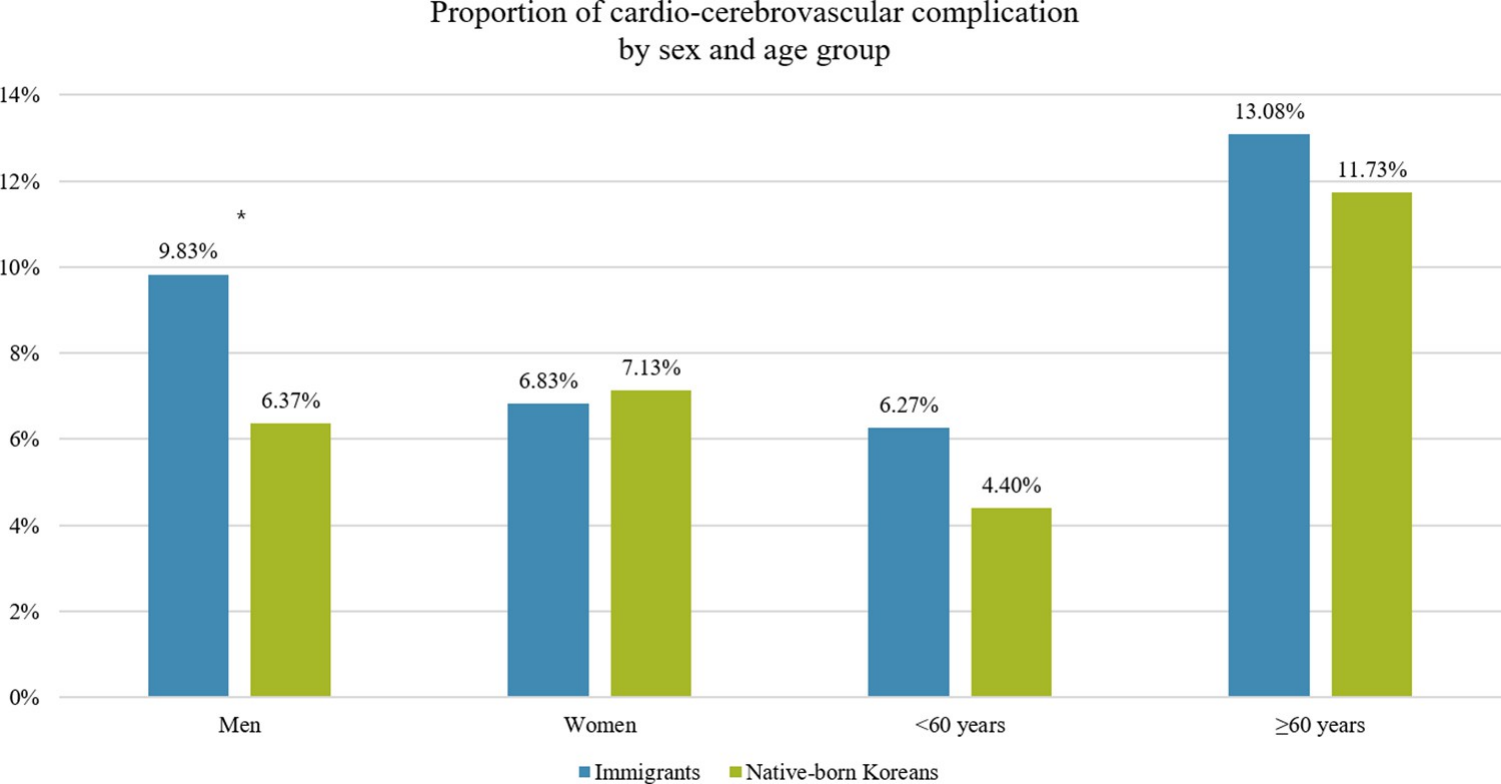
Proportion of cardio-cerebrovascular complications by sex and age group in both immigrants and native-born Koreans.

Fig 2 represents the comparison of CCV incidence by age and sex at 3-year follow-up between the subjects with diabetes in immigrants and native-born Korean not adjusting any covariates. Age, sex and CCI were originally matched at the first step of subject selection. As a whole, CCV incidences were higher in immigrant men (9.8%) compared to Korean men (6.37%) and this result was statistically significant (p = 0.0272). Whereas, immigrant women (6.8%) showed lower rate of CCV than Korean women (7.1%) and this was not significant. In age group comparison, immigrants showed higher CCV incidence in both <60 and ≥60 groups but both were not significant.

Sex and age stratified subgroup analyses were conducted for the risk of CCV complications in the two groups. Immigrant men showed significantly increased risk of CCV complications compared to Korean men (HR: 1.631, 95% CI: 1.089–2.444). In female and age comparisons, immigrants showed increased risks but with lack of significance (Table 3).

**Table 3:** Differences in cardio-cerebrovascular complications between immigrants and native-born Koreans by sex and age.

Category	Reference	CCV complication	
		HR	95% CI
**Male immigrants** ^†^	**Male native-born Koreans**	**1.631**	**1.089–2.444**
Female immigrants^†^	Female native-born Koreans	1.16	0.721–1.867
<60-year-old immigrants^‡^	<60-year-old native-born Koreans	1.447	0.940–2.228
≥60-year-old immigrants^‡^	≥60-year-old native-born Koreans	1.348	0.868–2.094

* CCV; Cardio-cerebrovascular, CCI; Charlson Comorbidities Index, MPR; Medication possession rate, USC; Usual source of care.

^**†**^Cox regression model with time to each complication (event), adjusting for age, CCI score, MPR, USC, number of anti-diabetic medications, insulin prescription at the first diagnosis, insurance type, insurance premium tier, residential area, number of household members, disability status, hypertension, hyperlipidemia, and type of medical institution visited at the first diagnosis.

^**‡**^Cox regression model with time to each complication (event), adjusting for sex, CCI score, MPR, USC, number of anti-diabetic medications, insulin prescription at the first diagnosis, insurance type, insurance premium tier, residential area, number of household members, disability status, hypertension, hyperlipidemia, and type of medical institution visited at the first diagnosis.

Table 4 shows that the risk factors for CCV complications in immigrants were evaluated. Age (≥60 years) and a high CCI score (1+) were significant risk factors, with age ≥ 60 years showing an HR of 2.5 (95% CI: 1.389–4.478) and a CCI score of 1+ having an HR of 1.9 (95% CI: 1.054–3.534). Immigrants who had a USC presented a significantly reduced risk of CCV complications (HR: 0.35, 95% CI: 0.175–0.703).

**Table 4:** Factors that affected the occurrence of cardio-cerebrovascular complications in immigrants.

Category		Reference	CCV complications	
			HR	95% CI
Male		Female	1.62	0.929–2.827
**≥60 years old**		**< 60 years old**	**2.494**	**1.389–4.478**
**CCI score ≥ 1**		**CCI score: 0**	**1.930**	**1.054–3.534**
Permanent resident		Naturalized	0.78	0.383–1.589
Marriage immigrant		Naturalized	0.973	0.466–2.029
MPR (continuous)			1.009	0.999–1.020
**USC (yes)**		**No**	**0.35**	**0.175–0.703**
Anti-diabetic medications: ≥2		Anti-diabetic medications: < 2	1.412	0.778–2.564
insulin prescription at the first diagnosis		Non-insulin prescription	0.918	0.353–2.385
Number of household members: 1		Number of household members: >1	0.95	0.526–1.716
Insurance premium tier: 5–20		Insurance premium tier: 0–4	1.22	0.638–2.336
Hypertension (yes)		No	0.969	0.502–1.871
Medical institution visited at the first diagnosis	Tertiary hospital	Clinic/public health center	1.732	0.584–5.135
	Secondary hospital	Clinic/public health center	1.376	0.691–2.74
	Primary hospital	Clinic/public health center	0.783	0.301–2.04

*CCV; Cardio-cerebrovascular, CCI; Charlson Comorbidities Index, MPR; Medication possession rate, USC; Usual source of care.

†Insurance type, hyperlipidemia, and disability were not compared due to their low proportions in immigrants.

## Discussion

We compared differences in the risk of CCV complications related to diabetes between immigrants and native-born Koreans and evaluated the risk factors for CCV complications among the immigrants. Adjusting for potential multiple covariates, the risk of CCV complications was 1.39 times higher in immigrants than in native-born Koreans. Further, we analyzed immigrants and native-born Koreans by age and sex categories and found that immigrant men had 1.63 times increased risk of CCV complications than Korean men when adjusting for age, CCI score, MPR, USC, number of anti-diabetic medications, insurance type, residential area, number of household members, disability status, hypertension, hyperlipidemia, and type of medical institution.

The risk factors for complications among immigrants were assessed. As immigrants became older and had higher CCI scores, they became more vulnerable to diabetes complications. In contrast, when immigrants had a consistent USC, their risk was significantly reduced by 64~70%.

Our findings are comparable to those of previous studies. Immigrants with diabetes have been found to have higher risks of mortality and complications than native-born residents in several studies 2. Klatsky et al. and Jose et al. reported that Asian-Americans with diabetes had a higher incidence of cerebrovascular diseases than non-immigrant Americans [41,42]. Fernando et al. found that South Asian migrants with diabetes had 1.5–2 times greater prevalence of coronary artery disease than Europeans [43].

Whereas Okrainec et al. [44]. and Franch-Nadal et al. [15] reported the controversial result that more immigrants failed to maintain optimal blood glucose levels than native residents, but had lower levels of diabetic complications. They speculated that the underlying reason for these findings may have been that the “healthy immigrant effect” persisted, resulting in a low prevalence of diabetic complications in immigrants despite poor blood glucose control. As another possible explanation, they also proposed that if immigrants confront new onset diabetes or become physically disabled to the point that they cannot cope with new challenges in a foreign country, they may return to their home country. Therefore, the prevalence of diabetes might have been accurately captured in their study, but long-term complications might have been underrepresented [15,44]. Therefore, long-term health care outcomes in immigrants might have been misestimated.

In our study, male immigrants showed significantly higher incidence of CCV complications than Korean male patients. Ku [45] reported that immigrants had less utilization of health care service than native residents due to the cost issue. Usually, male patients show low utilization of healthcare service than female, the low rate of diabetes management in male immigrants in this study is in line with the previous studies [46,47]. Other potential risk factors of CCV complication might include occupation related factors such as hard physical labor or stress related work, but we could not include these factors in this study, which might warrant the further study.

Potential risk factors for CCV complications in immigrants with diabetes were age (≥60 years old) and high CCI scores, which are well-known risk factors for CCV complications outcomes in general. However, having a USC significantly decreased incidence of diabetes complications in immigrants, which provides considerable insight into healthcare management and healthcare policy-making. A Dutch study investigated glucose control in Turkish immigrants and native-born residents and reached a similar conclusion that not having a consistent USC resulted in failed blood glucose management in Turkish immigrants [16]. Furthermore, a narrative review on diabetes management in South Asia pointed out the importance of strengthening the primary care model of care for preventing chronic complications [48]. In our previous study, we also ascertained that whether to have USC significantly determined medication compliance in immigrants in highly prevalent chronic hypertension management [49].

Additionally, even though the results were not significant, a high MPR was associated with an increased risk of CCV complications, as were a high number of anti-diabetic medications (≥2) at the first prescription and receiving that prescription at higher-level medical institutions. These results align with those of previous studies reporting that patients with CCV complications tended to have better drug adherence [50], and they might reflect the tendency of patients with severe diabetes to be more likely to have a high number of prescription drugs and to visit or be referred to higher-level medical institutions. Hypertension has been pointed out as a significant risk factor for macrovascular events, with a 1.753-fold increased risk in 702 cerebral infarction patients [51]. However, in our study, hypertension was not found to be a significant risk factor for cardio-cerebrovascular complications.

Other factors also need to be considered, even if they are beyond the scope of our study. Monetesi et al. proposed that differences in macrovascular complications between immigrants and native residents were attributable to genetic predisposition, cultural and diet-related lifestyle changes after immigration, socioeconomic factors (labor environment, education level, and low income), language barriers, high medical costs, and low healthcare literacy [17]. Testa et al. also suggested that differences in genetic profiles, lifestyle, and utilization of the health care system could affect the incidence of complications and mortality of diabetes [52]. Further studies incorporating these factors will be needed for a more comprehensive and systematic analysis.

This study has a few strengths worth mentioning. This is the first study to analyze differences in the risk of CCV complications between immigrants and native-born Koreans with diabetes using administrative data. Through this study, we confirmed that immigrants with diabetes are more vulnerable to CCV complications and discovered unmet needs in this specific population. We also identified some risk factors and protective factors associated with CCV complications among immigrants. In addition to our findings regarding non-modifiable factors such as aging and CCI scores, we found a very important modifiable factor—USC. This finding can be utilized to further advance healthcare policy aiming to promote quality of life, well-being, and safe acculturation among immigrants. We applied well-developed criteria to define patients with diabetes and CCV complications with the help of specialists in endocrinology, which is a further strength of this study that enabled false positives to be removed to the extent that is possible with secondary data.

We also need to address a few limitations of this study. First, we used the KNHICD, a secondary data source with the inherent limitation of only capturing data from records of patients’ visits covered by health insurance. Therefore, small number of immigrants included in this study might be explained by this aspect. A considerable part of immigrant who were not eligible for insurance coverage in study period should have been omitted. Ethnic factor might be one of important risk factor of diabetic complications but most immigrants included in this study were naturalized Korean, so we could not retrieve and evaluated their country of origin in our database. Second, KNHICD contains electronic medical records for over 97% of the entire population but basically, claim data is generated to claim insurance coverage reimbursement, so sometimes using the diagnosis or procedure codes to ascertain research subjects might include false positive subjects. In the awareness of this limitation, we used more stringent criteria such as subjects who had diabetes ICD-10 codes as primary to the fifth level diagnosis code as well as prescriptions for anti-diabetic medications. In addition, patients who had tow and more outpatient clinic visits, or one and more inpatient department visits with diabetes ICD-10 were included. On the other hand, due to the limitation of the database, if patients had already developed CCV complications at their initial diagnosis of diabetes, the outcomes of CCV complications could not be included in this analysis, which might have caused the outcomes to be underestimated. Third, due to the characteristics of insurance claim data, lab data such as HbA1c or serum glucose level, anthropometric data such as BMI or obesity, health related behavior data such as diet or physical activity, psychosocial stress, smoking history data as well as family history data which might be potential covariates of CCV could not be evaluated. Therefore, the severity of diabetes could not be assessed. However, we included the number of antidiabetics medication prescribed at the diagnosis, medical institution that first issued anti-diabetic prescription, and insulin prescription at the first diagnosis to complement this issue. Insulin prescription can be a factor to differentiate diabetes severity but the small number of immigrants having insurance prescription precluded a further analysis. In addition, the comparison on insulin prescription at the first diagnosis was not significant between the two groups. Also, we need to mention that we couldn’t differentiate the complex antidiabetic medications containing 2–3 agents in a pill from single agent. Also compared to the nature of long-term CCV outcomes, the follow-up period was short. Fourth, we used the MPR as a proxy index for drug compliance, although it might not reflect real drug adherence (as would be possible through pill counting) and overestimate the drug compliance rate. Last, we could not define continuity of care or incorporate this term into our study; instead, we analyzed USC to obtain an approximate glimpse of continuity of care.

## Conclusion

This study found a higher risk of CCV complications in immigrants with diabetes than in native-born Koreans with diabetes. However, having a USC significantly decreased the risk of CCV complications. Therefore, the utilization of USC will benefit to reduce diabetic complications in immigrants as well as reduction of overall health care cost burden, it would be necessary to implement USC in diabetes care at the initial disease stage.
